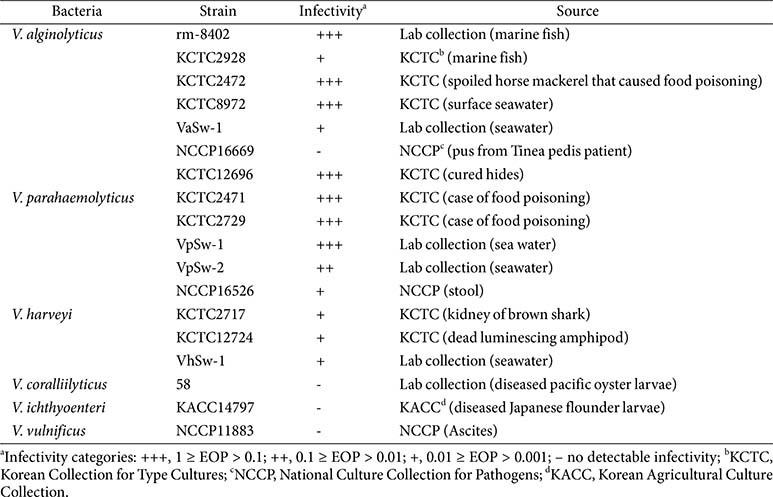# Antivirulence Activity and Therapeutic Potential of Jumbo *Vibrio* Phage pVa-22 against *Vibrio alginolyticus*

**DOI:** 10.4014/jmb.2509.09037

**Published:** 2025-11-27

**Authors:** Su Jin Jo, Jun Kwon, Se Chang Park, Sang Guen Kim

**Affiliations:** 1 Laboratory of Aquatic Biomedicine, College of Veterinary Medicine and Research Institute for Veterinary Science, Seoul National University, Seoul 08826, Republic of Korea; 2 Laboratory of Veterinary Public Health, College of Veterinary Medicine, Jeonbuk National University, Iksan 54596, Republic of Korea; 3 Laboratory of Phage and Microbial Resistance, Department of Biological Sciences, Kyonggi University, Suwon 16227, Republic of Korea

**Keywords:** *Vibrio alginolyticus*, jumbo bacteriophage, phiKZ-like phage, *Galleria mellonella*

## Abstract

*Vibrio alginolyticus* is an emerging zoonotic pathogen responsible for severe aquaculture and human infections, and its increasing antimicrobial resistance calls for alternative control strategies. Here, we report the isolation and characterization of the jumbo phage pVa-22, a phiKZ-like phage that infects *V. alginolyticus*. Morphological analysis of the phage via transmission electron microscopy revealed a large icosahedral capsid and contractile tail consistent with jumbo vibriophages. pVa-22 exhibited notable stability across broad range of temperature (4–37°C) and pH values (3–11), as well as rapid adsorption and a moderate burst-size and latent period. Host range assays revealed strong lytic activity against *Vibrio* species associated with foodborne illness, including *V. alginolyticus* and *V. parahaemolyticus*. Genomic analysis revealed a 233,667-bp double-stranded DNA genome encoding 242 predicted open reading frames, including multisubunit RNA polymerases and putative phage nucleus-associated proteins, which are characteristic of phiKZ-like jumbo phages. Comparative genomics demonstrated close pVa-21 relatedness but divergence in regions linked to nucleotide metabolism, tail fibers, and replication-associated genes. Functionally, pVa-22 showed weak bactericidal effects *in vitro*; however, it significantly enhanced larval survival in a *Galleria mellonella* infection model, suggesting antivirulence activity. Putative PadR-type transcriptional regulators encoded by pVa-22 may potentially influence quorum-sensing pathways, although experimental validation is required. Taken together, the findings revealed that pVa-22 is a jumbo vibriophage possessing antivirulence activity and therefore therapeutic potential, expanding the repertoire of phage-based therapeutic strategies against climate-driven *Vibrio* outbreaks.

## Introduction

*Vibrio alginolyticus* is an emerging zoonotic pathogen that has shown increasing significance in marine and human health [1]. This halophilic bacterium, belonging to the Harveyi clade, is widely distributed in coastal environments and has been implicated in a broad range of infections [2]. In marine animals, *V. alginolyticus* causes systemic diseases in fish, shrimp, and mollusks, frequently leading to mass mortality events in aquaculture [3, 4]. In humans, exposure to seafood or seawater contaminated with *V. alginolyticus* can result in gastroenteritis, otitis, and wound infections, particularly in immunocompromised individuals [5]. In recent years, the incidence of *V. alginolyticus* infections has risen globally, a trend that is thought to be linked to climate change [6]. Rising sea surface temperatures and longer warm seasons create favorable conditions for the proliferation, virulence, and geographic expansion of *Vibrio* species, positioning *V. alginolyticus* as a growing concern [7].

The threat of *V. alginolyticus* is compounded by its capacity to acquire and disseminate antimicrobial resistance (AMR) [8]. Strains isolated from aquaculture and clinical environments have demonstrated resistance to multiple classes of antibiotics, including β-lactams, tetracyclines, and fluoroquinolones [4, 9, 10]. The misuse of antibiotics in aquaculture has accelerated the selection of resistant populations, thereby diminishing the effectiveness of conventional treatments and raising biosecurity concerns [11]. *V. alginolyticus* can serve as a reservoir for AMR genes, facilitating horizontal gene transfer to other *Vibrio*s and clinically relevant pathogens [12]. These challenges highlight the urgent need for alternative approaches for the control of *V. alginolyticus*, especially in the context of global warming, where the pathogen’s prevalence and AMR are likely to intensify.

Lytic bacteriophages are promising alternatives to antibiotics due to their advantages of self-amplification at the infection site, minimal disruption of the microbiota, and adaptability to bacterial resistance [13]. Several *V. alginolyticus*-targeting phages have been reported, including jumbo phages with large genomes that encode their own transcriptional systems and form protective phage nuclei [14-16]. Notably, jumbo phages infecting *Vibrio* have demonstrated strong lytic activity, stability under aquaculture-relevant conditions, and therapeutic efficacy *in vivo* [17-19]. In addition to traditional lytic effects, recent attention has turned to antivirulence phages that protect hosts by suppressing bacterial virulence factors rather than eliminating bacterial populations [20]. Such an approach may provide a more ecologically balanced and evolutionarily stable means of disease control. However, comprehensive information regarding their biological and genomic characteristics remains limited. Expanding the vibriophage library is essential for effective phage-based strategies for the biological control of *V. alginolyticus* in aquaculture and public health.

Despite growing reports of jumbo vibriophages, their antivirulence potential and comparative genomic diversity remain poorly characterized. To address these gaps, we isolated and characterized a novel vibriophage pVa-22, and elucidated its biological and genomic traits, as well as its *in vivo* therapeutic activity using a *Galleria mellonella* infection model. These findings support the potential of jumbo phages as effective biocontrol agents against climate-driven *Vibrio* outbreaks in aquaculture and beyond.

## Materials and Methods

### Bacterial Culture Conditions and Bacteriophage Isolation

Phages infecting the pathogenic *V. alginolyticus* rm-8402 were isolated following the method described previously by Kim *et al*. [21]. To enrich the phages, exponentially growing *V. alginolyticus* cultures were mixed with environmental water samples and incubated at 27°C for 24 h. The cultures were centrifuged, and the supernatants were serially diluted and spotted onto bacterial lawns to detect phage activity. Plaque-containing samples were filtered through a 0.2-μm membrane to remove residual bacteria. The presence of lytic phages was confirmed via double-layer agar assay. To obtain clonal phage stocks, individual plaques were purified through five rounds of single-plaque isolation.

### Bacteriophage Preparation

Phage propagation and purification were performed as described previously [22]. The top agar layers containing phage plaques were harvested in sodium–magnesium (SM) buffer (50 mM Tris-HCl, pH 7.5; 100 mM NaCl; 10 mM MgSO_4_) and homogenized. The mixture was centrifuged at 10,000 ×*g* for 20 min, and NaCl was added to the supernatant to a final concentration of 0.5 M. After an additional centrifugation step, polyethylene glycol 8000 was added to precipitate the phage particles. The resulting phage pellet was further purified by cesium chloride gradient ultracentrifugation (50,000 ×*g* for 3 h). The visible phage band was collected and dialyzed against SM buffer using a dialysis cassette (7,000 MWCO, Slide-A-Lyzer, Thermo Scientific, USA), and the purified phage solution (>10^10^ plaque-forming units [PFU]/ml) was stored at 4°C until further use.

### Transmission Electron Microscopy

The morphological features of phage pVa-22 were examined under transmission electron microscopy (TEM) as described previously [23]. In brief, the purified virions were adsorbed onto glow-discharged carbon-coated copper grids for 1 min. The excess liquid was removed, and the grids were stained with 2% (w/v) uranyl acetate for negative staining. After removing the stain and air-drying, the grids were examined under a TEM (Talos L120C; FEI, USA) operated at 120 kV. Representative images were used to measure the phage dimensions, and the average values were calculated from three independent virions.

### Bacteriophage Host Range Assay

The host range of phage pVa-22 was assessed using a panel of 18 *Vibrio* strains, which comprised seven *V. alginolyticus* strains, five *V. parahaemolyticus* strains, three *V. harveyi* strains, one *V. coralliilyticus* strain, one *V. ichthyoenteri* strain, and one *V. vulnificus* strain. The strains were isolated from marine environment, diseased marine animals and clinical case. The phage infectivity was evaluated using spot assays with serial dilutions of each phage lysate on bacterial lawns. The efficiency of plating (EOP) was determined as the ratio of plaque numbers on a test strain to those on the reference host strain (rm-8402). Infectivity was categorized as follows:+++, 1 ≥ EO*P* > 0.1; ++, 0.1≥EO*P* > 0.01; +, 0.01 ≥ EO*P* > 0.001; and –, no detectable infectivity. The EOP values were used to estimate the relative infectivity and lytic capacity of each phage across the different *Vibrio* species. To ensure reproducibility, all experiments were performed in triplicate.

### Adsorption Assay

The adsorption kinetics of phage pVa-22 were assessed following a modified protocol described by Kim *et al*.[24]. In brief, exponentially growing host cells (2 × 10^8^ CFU/ml) were mixed with phages at a multiplicity of infection (MOI) of 0.001 and incubated at 27°C. At defined time intervals (0, 1, 3, 5, 7, 10, 15, 20, 30, 40, and 50 min), 100-μl samples were collected, diluted 10-fold in PBS, and centrifuged at 12,000 ×*g* for 5 min. The concentration of free, unadsorbed phages in the supernatant was then determined using the double-layer agar method. To ensure reproducibility, all experiments were performed in triplicate.

### One-Step Growth Assay

A one-step growth experiment was conducted to determine the replication kinetics of the phages, as previously described [24]. In brief, a 100-μl aliquot of the phage suspension was added to 10 ml of exponentially growing bacterial culture (2 × 10^8^ CFU/ml) at an MOI of 0.001. After incubation for 50 min at 27°C to ensure sufficient phage adsorption, the mixture was centrifuged at 12,000 ×*g* for 2 min to remove unadsorbed phages. The pellet containing phage-infected cells was resuspended in 10 ml of prewarmed broth and incubated at 27°C with shaking at 150 rpm. Aliquots were collected every 10 min for 140 min, and the double-layer agar method was used to determine the phage titers. To ensure reproducibility, all experiments were performed in triplicate.

### DNA Isolation

Phage genomic DNA was extracted using a modified phenol–chloroform protocol [25]. In brief, 1 ml of highly concentrated phage suspension (10^10^ PFU/ml) was treated with 10 U each of RNase A and DNase I to degrade any contaminating host nucleic acids. The reaction mixture was incubated at 37°C for 1 h, followed by the addition of 0.5 M EDTA to halt the nuclease activity. Proteinase K was added to digest the capsid proteins, and the sample was incubated at 56°C for 1 h. For DNA extraction, an equal volume of phenol:chloroform:isoamyl alcohol (25:24:1, v/v) was added, thoroughly mixed with the sample, and the samples were centrifuged at 4°C for 10 min. The aqueous phase was carefully collected, 0.3 M sodium acetate (pH 5.2) was added, and the genomic DNA was precipitated by adding 2.5 volumes of ice-cold 99% ethanol. The DNA pellet was washed twice with 70% ethanol, air-dried, and resuspended in nuclease-free water for the downstream applications.

### Sequencing and Genome Analysis

Whole-genome sequencing of phage pVa-22 was performed at Macrogen (Republic of Korea) using the Illumina HiSeq 2500 platform (Illumina, USA). Genomic libraries were prepared using the Illumina TruSeq Nano DNA Library Prep Kit according to the manufacturer’s instructions. Sequencing achieved an average coverage depth of approximately 2,170×, confirming the high quality of the whole-genome sequence. Raw sequencing reads were quality-trimmed using Trimmomatic (v0.36) to remove low-quality bases and adapters [26]. The resulting high-quality reads were assembled *de novo* using SPAdes (v3.12) [27]. Gene prediction and annotation were performed using multiple tools, including Prokka (v1.12b) [28], GenMarkS [29], RAST [30], BLASTp, and HHpred [31]. tRNAscan-SE (v2.0) was used to identify transfer RNA genes [32]. Whole-genome dot plot comparisons, phylogenetic trees, and comparative analysis were constructed using Gepard [33], the Virus Classification and Tree Building Online Resource [34], and clinker [35].

### Antibacterial Tests

The antibacterial effect of phage pVa-22 was evaluated using *V. alginolyticus* rm-8402 as the indicator strain as previous description [14]. Bacterial cultures in the exponential phase (2 × 10^7^ CFU/ml) were inoculated with pVa-22 at different MOIs (0.1, 1, and 10), then incubated at 27°C with shaking at 150 rpm. Bacterial growth was monitored by measuring the optical density at 600 nm (OD_600_) at regular intervals over a 24-h period. To ensure consistency and reproducibility, all assays were performed in triplicate. Differences in OD_600_ among treatment groups were evaluated using one-way ANOVA, with *P* < 0.05 considered statistically significant.

### *V. alginolyticus* Infection in the *Galleria mellonella* Model

To assess the virulence of *V. alginolyticus* rm-8402, healthy *Galleria mellonella* larvae (100–130 mg) were maintained at room temperature without feeding for 24 h prior to challenge. Larvae were orally inoculated with serial dilutions of bacterial suspensions (10^6^–10^3^ CFU/ml) harvested at the early log phase, washed with sterile phosphate-buffered saline (PBS), and resuspended prior to inoculation. The control group received sterile PBS. Each group contained 10 larvae and was tested in triplicate. Larvae were randomly assigned to treatment groups and survival assessment was conducted in a blinded manner. The larvae were incubated at 27°C and monitored every 24 h for 120 h. Mortality was defined as the absence of movement following tactile stimulation. The 90%lethal dose (LD_90_) was calculated by linear regression analysis of the dose-response curve and defined as the bacterial inoculum that caused 90% mortality in *G. mellonella* larvae.

The therapeutic efficacy of phage pVa-22 against *V. alginolyticus* was assessed using a model of *G. mellonella* infection challenged at the LD_90_ dose. Healthy larvae (100–130 mg) were acclimated at room temperature without feeding for 24 h prior to the experiment. For prophylactic treatment, larvae were orally administered with 10 μl of pVa-22 at concentrations ranging from 2 × 10^6^ to 2 × 10^8^ PFU/ml. After 1 h, 10 μl of the bacterial suspension at the LD_90_ dose was orally administered to the larvae. The control groups received sterile SM buffer or PBS. The larvae were incubated, and the mortality was assessed based on the absence of movement in response to gentle stimulation. Survival differences among treatment groups were evaluated using the log-rank test, with *P* < 0.05 considered statistically significant.

### Nucleotide Sequence Accession Number

The complete genome sequence of phage pVa-22 was deposited in GenBank under accession number PX097379.

## Results and Discussion

### Biological Characterization of the pVa-22 Jumbo Phage

**Fig. 1:**
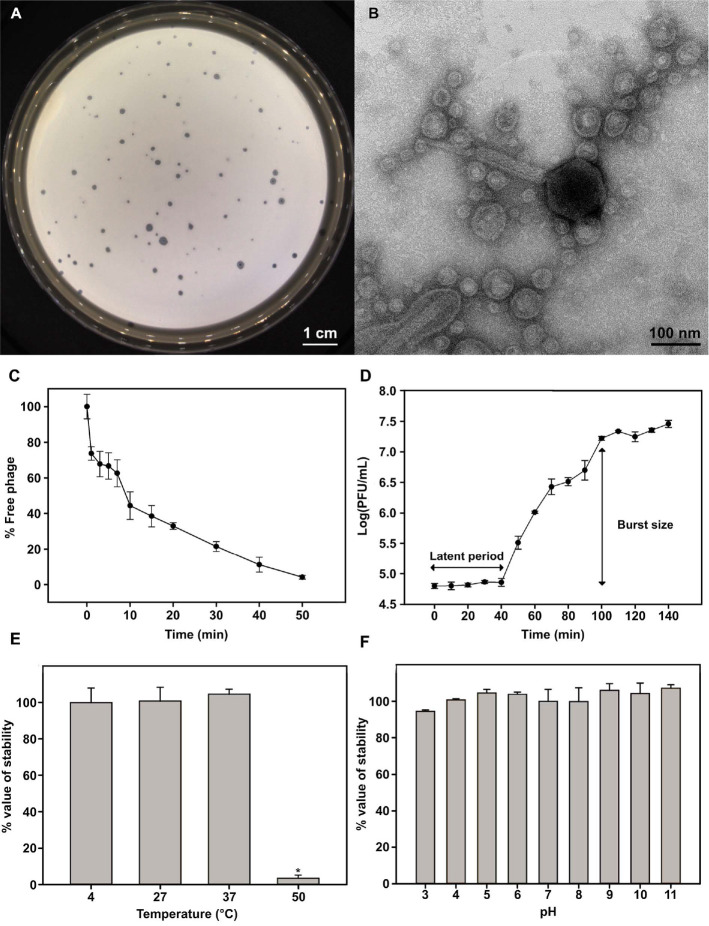
Biological characteristics of the jumbo phage pVa-22. (**A**) Plaque morphology of pVa-22 on *Vibrio alginolyticus* rm-8402. (**B**) Transmission electron micrograph showing phage morphology. (**C**) Adsorption kinetics and (**D**) one-step growth curve of pVa-22 illustrating the latent period and burst size. pVa-22 stability under different temperature (**E**) and pH conditions (**F**). Data represent mean ± standard deviation from triplicate experiments.

The plaque morphology of pVa-22 was characterized using lawns of *V. alginolyticus* rm-8402. After 24 h of incubation at 27°C, the phage produced clear, round plaques with diameters of approximately 1–2.5 mm, indicating strong lytic activity toward its primary host. No turbid halos were observed, suggesting the absence of lysogeny or depolymerase activity under the tested conditions (Fig. 1A).

TEM revealed that phage pVa-22 possesses an icosahedral head measuring approximately 95 ± 2.2 nm in diameter and a contractile tail of 246 ± 4.5 nm in length (*n* = 10) (Fig. 1B). Its closest relative, pVa-21, exhibited a comparable head (87 nm) and tail length (240 nm), suggesting structural conservation and subtle morphological divergence between the two phages. The head and tail dimensions of pVa-22 fall within the established range of other jumbo vibriophages. For example, members of the genus *Schizotequatrovirus* display prolate capsids of 120–130 nm and contractile tails of approximately 110 nm in length (*e.g.*, PVA23 with a 134 nm head and 115 nm tail; F86 with a 125 nm head and 110 nm tail) [19, 36], members of the subfamily *Gorgonvirinae*, such as phiKT1019 (104.57 ± 7.31 nm head, 226.63 ± 12.50 nm tail) and phiKT1028 (129.25 ± 2.7 nm head, 232.35 ± 2.30 nm tail) [37], and the vibriophage Yong-XC31 (head ~113 nm, tail ~219 nm) [38], exhibit similar dimensions. The relatively larger head size of pVa-22 is consistent with its genome size of >230 kb, reflecting the capsid expansion required to package jumbo phage genomes.

Adsorption kinetics demonstrated the rapid attachment of pVa-22 to its host, *V. alginolyticus* rm-8402, with approximately 50 % of phages adsorbed within the first 10 min and near-complete adsorption observed by 50 min (Fig. 1C), which were comparable to its close relative pVa-21. A one-step growth experiment revealed a latent period of 40 min, followed by a growth period of 60 min, yielding an average burst size of approximately 450 PFU per infected cell (Fig. 1D). While comparable to other jumbo vibriophages, the smaller burst size of pVa-22 relative to its closest relative, pVa-21, likely contributes to its limited bactericidal effect in planktonic cell lysis assays [14, 18, 19, 36].

To assess its suitability for therapeutic and environmental applications, the stability of pVa-22 was examined under different physicochemical conditions. The phage retained infectivity after incubation at temperatures ranging from 4°C to 37°C but exhibited a significant reduction in activity at 50°C (Fig. 1E). Similarly, it remained stable across a broad pH range (pH 3–11) (Fig. 1F). These findings indicate that pVa-22 possesses considerable environmental robustness, an important trait for its application in aquaculture and clinical contexts.

The host range of pVa-22 was evaluated against 18 *Vibrio* strains, including seven *V. alginolyticus* isolates and representatives of five additional species and infectivity was categorized based on EOP values (Table 1). Phage pVa-22 exhibited broad lytic activity against pathogenic and foodborne illness–associated *Vibrio* species within the Harveyi clade, including *V. alginolyticus*, *V. harveyi*, and *V. parahaemolyticus*, similar to other jumbo vibriophages. However, it showed no infectivity toward other species such as *V. coralliilyticus*, *V. ichthyoenteri*, and *V. vulnificus* (Table 1).

### Genomic Characterization of the pVa-22 Jumbo Phage

Illumina sequencing of phage pVa-22 yielded 724,947,664 raw base pairs with an average coverage of 2,170×, ultimately assembling into a single contig of double-stranded DNA. The complete genome of pVa-22 was 233,667 bp in length, with a GC content of 44.54%, thereby classifying it as a jumbo phage. Genome annotation identified 242 CDSs and no tRNAs (Table S1). These features are highly comparable to those of its closest relative, phage pVa-21, which harbors a genome of 231,998 bp with 241 CDSs and a 44.58% GC content [14]. The absence of tRNAs in both genomes suggests reliance on the host’s translational machinery, a feature that might contribute to its host specificity and slower replication kinetics compared with other jumbo phages [39]. In contrast, other jumbo phages generally encode multiple tRNAs to optimize translation efficiency during infection.

The functional annotation of pVa-22 revealed a similar modular organization as that observed in pVa-21, including structural proteins, DNA replication and repair enzymes, transcriptional regulators, and host takeover proteins. Notably, pVa-22 encodes multi-subunit RNA polymerases and putative phage nucleus-associated proteins, characteristic features of phiKZ-like jumbo phages (Table S1) [14, 18, 37, 40]. These genetic hallmarks indicate that pVa-22 likely establishes a subcellular compartment resembling a phage nucleus during infection, thereby protecting viral DNA from host nucleases and enhancing transcriptional autonomy.

**Fig. 2:**
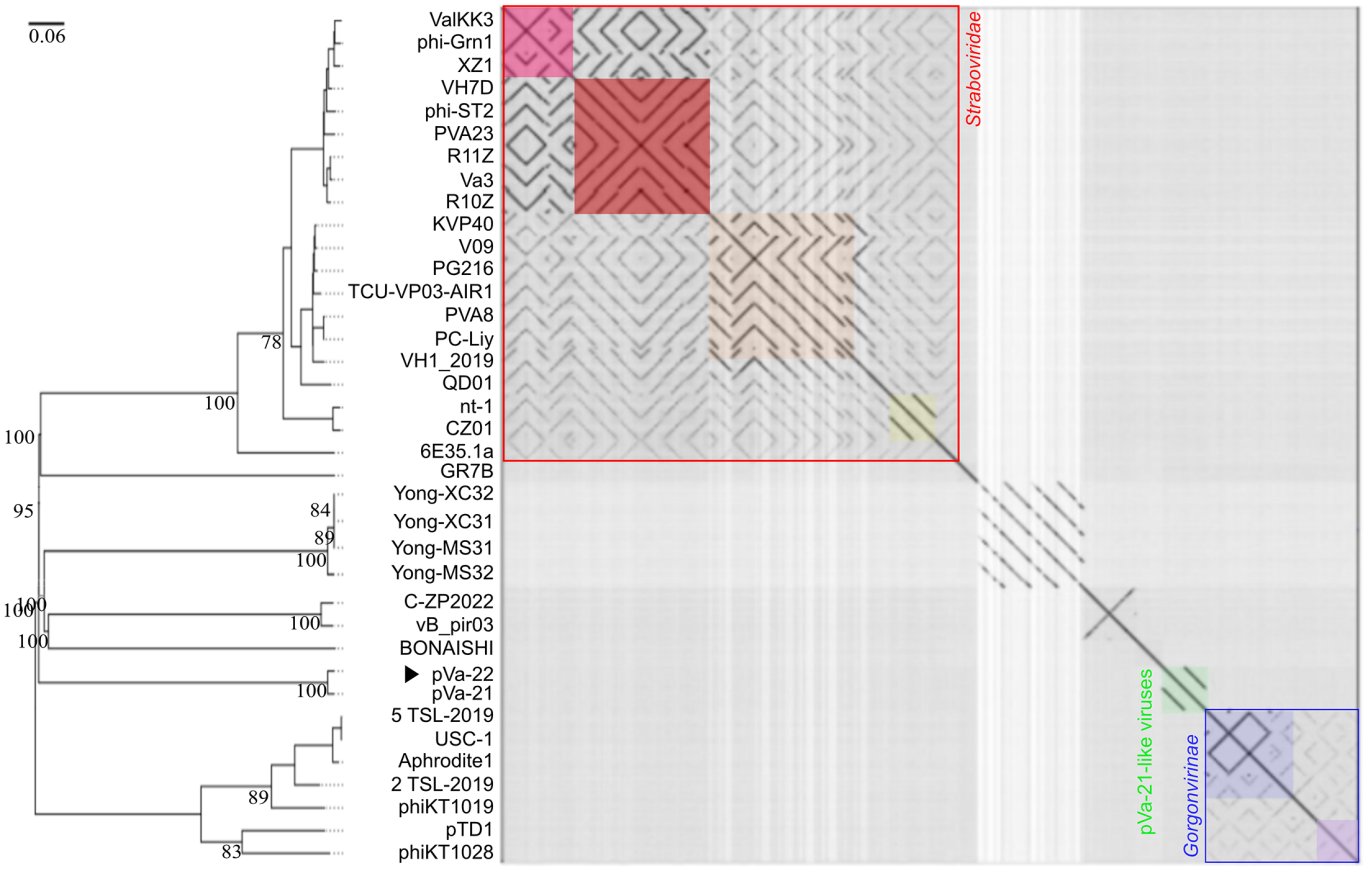
Whole-genome phylogenetic and dot plot analyses of pVa-22 and jumbo vibriophages. The phage isolated in this study is indicated by the arrow (▶). Genera are distinguished by color coding: *Schizotequatrovirus* ValKK3 (pink box, family *Straboviridae*), *Schizotequatrovirus* Vh7D (red box, family *Straboviridae*), *Schizotequatrovirus* KVP40 (orange box, family *Straboviridae*), *Mylasvirus* (yellow box, family *Straboviridae*), pVa-21-like viruses (green box), *Aphroditevirus* (blue box, subfamily *Gorgonvirinae*), and *Tidunavirus* (purple box, subfamily *Gorgonvirinae*).

Comparative genomic analysis demonstrated a very close relationship between pVa-22 and pVa-21 (Fig. 2). A BLASTn search revealed that pVa-22 shared 98% coverage with pVa-21, with 99.35% identity. Both phages displayed only limited sequence similarity to other jumbo vibriophages, including members of *Schizotequatrovirus* (ValKK3, phi-Grn1, XZ1, VH7D, phi-ST2, PVA23, R11Z, Va3, R10Z, KVP40, V09, PG216, TCU-VP03-AIR1, PVA8, PC-Liy1, VH1-2019), *Mylasvirus* (nt-1), and unclassified vibrio phages (QD01, CZ01, 6E35.1a) within the family *Straboviridae*. Additional low-level similarity was observed between *Aphroditevirus* members (5TSL-2019, USC-1, Aphrodite1, 2TSL-2019) and *Tidunavirus* representatives (pTD1, phiKT1028) belonging to the subfamily *Gorgonvirinae*. This distinct pattern of similarity suggests that the pVa-21/pVa-22 lineage forms a separate phylogenetic group of jumbo vibriophages. Dot plot comparisons confirmed the genomic distance of pVa-21-like viruses from other well-classified jumbo vibriophages, further supporting their unique evolutionary status (Fig. 2).

**Fig. 3:**
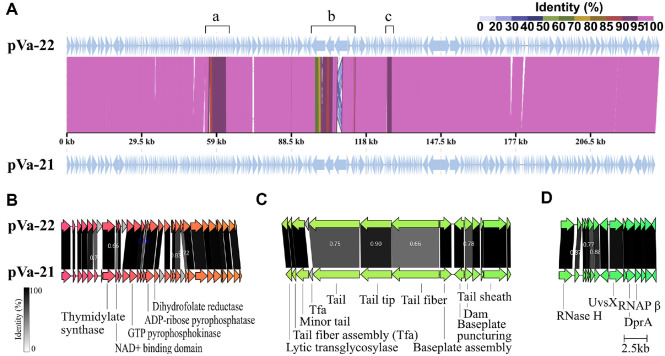
Comparative genomic analysis of jumbo phage pVa-22 with other jumbo vibriophages. (**A**) Wholegenome alignment and synteny comparison between pVa-22 and pVa-21. (**B**) Variable region “a” associated with nucleotide metabolism. (**C**) Variable region “b” encoding tail-associated proteins. (**D**) Variable region “c” encoding nucleic acid processing proteins.

Whole-genome synteny analysis was conducted to explore the genome-level differences between pVa-21 and pVa-22, which showed extensive collinearity with pVa-21 (Fig. 3A) [14]. Three divergent regions, designated “a–c,” were identified and further analyzed (Fig. 3B–3D). Region “a” contained genes related to nucleotide metabolism, including thymidylate synthase, GTP pyrophosphokinase, ADP-ribose pyrophosphatase, and dihydrofolate reductase (Fig. 3B). Several hypothetical proteins (gp56, gp59, gp65, gp70, and gp73 in pVa-22) displayed <90%amino acid identity with their homologs in pVa-21. Furthermore, pVa-22 lacked two genes (gp67 and gp72) present in pVa-21, whereas pVa-22 contained a unique insertion (gp61), indicating local genomic rearrangements. These differences may alter the nucleotide biosynthesis efficiency during infection. Region “b” was associated with tail-related proteins, including tail fibers, tail, and tail tip proteins, which displayed high synteny but only moderate sequence identity (66%–90%) between the two phages (Fig. 3C). Divergence in these proteins, particularly tail fibers, is frequently linked to differences in host range. Comparative analysis with its close relative pVa-21 showed that pVa-22 possesses a broader host range [14]. This expanded spectrum likely reflects variations in tail fiber and other tail-associated proteins, which exhibited relatively low sequence identity between the two phages (Fig. 3C). These findings also provide valuable insight into the potential for phage engineering to broaden host range. The DNA adenine methyltransferase (Dam) gene differed substantially between the two phages (78%identity), suggesting variations in DNA modification and restriction evasion strategies, which could further influence host range specificity (Table S1). Region “c” encodes nucleic acid processing proteins, including RNase H, UvsX, DprA, and the RNA polymerase β subunit, a defining feature of phiKZ-like jumbo phages (Fig. 3D) [40]. Within this region, three hypothetical proteins (gp141, gp144, and gp147 in pVa-22) exhibited only 77%–88%identity with their counterparts in pVa-21. Such divergence may influence the replication dynamics. Consistent with this, the one-step growth experiments revealed that pVa-22 displayed a latent period of 40 min and a burst size of 450 PFU per infected cell, compared with 70 min and 860 PFU for pVa-21 (Fig. 1D). These phenotypic differences suggest that the functional divergence in the replication strategies of the two closely related phages may be attributed to genomic variations within region c.

The genomic characterization of pVa-22 highlighted both its close evolutionary relationship with pVa-21 and its unique genomic signatures. Although the overall gene content and synteny are highly conserved, functional diversification is suggested by divergence in regions associated with nucleotide metabolism, host range determination, and replication machinery. These features underscore the distinct evolutionary trajectory of pVa-22 within jumbo vibriophages and provide insights into the genetic basis of its biological properties.

### Antivirulent Therapy Using the pVa-22 Jumbo Phage

**Fig. 4:**
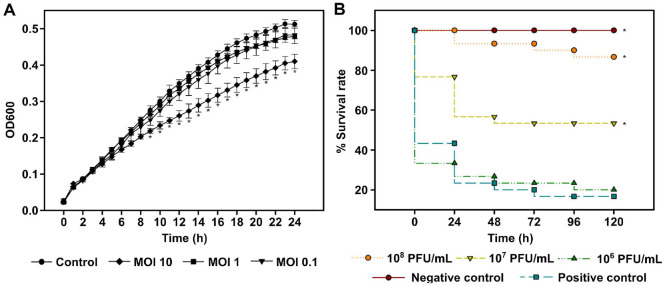
Antivirulence activity and therapeutic potential of the jumbo phage pVa-22. (**A**) Planktonic cell lysis assay of *V. alginolyticus* rm-8402 treated with pVa-22 at different multiplicities of infection (MOIs, 0.1–10). (**B**) Survival of *Galleria mellonella* larvae infected with *V. alginolyticus* at the 90%` lethal dose and treated with pVa-22. Survival was monitored for 5 days across triplicate experiments (*n* = 10 larvae per group).

The bactericidal potential of pVa-22 against *V. alginolyticus* rm-8402 was first examined *in vitro* using a planktonic cell lysis assay. Across a range of MOIs, pVa-22 did not produce a pronounced reduction in bacterial density, and no complete clearance was observed over the 24-h experimental period (Fig. 4A). Only a slight reduction in bacterial density was observed at the highest MOI (10) from 9 h post-inoculation, which was statistically significant (*P* < 0.05), although complete clearance was not achieved overall. This limited *in vitro* lytic effect contrasts with that of other jumbo phages, which demonstrate strong bactericidal activity under similar conditions [14, 16, 18, 19, 37, 41, 42]. For example, its closest relative, phage pVa-21, exhibited robust lytic efficacy at MOIs of 1 and 10, achieving complete clearance of the bacterial population, whereas a significant reduction in density was observed at an MOI of 0.1 [14]. Similarly, jumbo vibriophages such as PVA8, Grn1, and ST2 (members of *Schizotequatrovirus*) displayed dose-dependent bactericidal effects across MOIs ranging from 0.1 to 100 [41, 42]. In addition, although unclassified, the broad-host-range jumbo phage vB_VhaM_pir03 showed effective inhibition of bacterial growth at MOIs between 0.1 and 10 [18]. Collectively, these comparisons suggest that pVa-22 does not act primarily through direct population suppression in planktonic culture.

Despite this weak *in vitro* bactericidal activity, pVa-22 exhibited pronounced protective effects *in vivo* (Fig. 4B). In the *G. mellonella* infection model, larvae challenged with the 90% lethal dose of *V. alginolyticus* displayed significantly increased survival when treated with pVa-22 compared with untreated controls (Fig. 4B). At the LD_90_ challenge dose, 10^8^ PFU/ml conferred 85% survival and 10^7^ PFU/ml 45% survival, both of which were statistically significant (*P* < 0.05). In contrast, 10^6^ PFU/ml showed no effect compared with the untreated controls (*P* = 0.9414). These findings indicate that pVa-22 exerts therapeutic potential through antivirulence mechanisms rather than through wholesale bacterial eradication. While the *G. mellonella* larval model provides a cost-effective and ethically favorable system for preliminary evaluation of phage efficacy, as widely used in other studies, it does not fully replicate the physiological conditions of aquatic hosts. Therefore, further validation using fish or shellfish infection models is required to confirm the therapeutic relevance of pVa-22 in aquaculture settings.

The concept of phage-mediated bacterial virulence attenuation has gained increasing attention as a novel therapeutic strategy [42]. For instance, *Pseudomonas* phages carry the tip or moron genes, which impair twitching motility and reduce virulence [43, 44]. Similarly, the cell wall-binding domains of certain phages have been reported to neutralize bacterial surface components, such as the peptidoglycan of *Staphylococcus aureus* [45] or the lipopolysaccharide of *P. aeruginosa*, attenuating their pathogenicity [46]. Although the precise mechanism underlying the anti-virulence effects of pVa-22 remains unclear, this represents the first documented antivirulence phenotype in a jumbo vibriophage, with genomic features suggesting possible molecular clues (Table S1). Phage pVa-22 encode two putative PadR-type transcriptional regulators (gp6 and gp98). In *Vibrio* spp., AphA, a representative PadR-type regulator, acts as a master transcriptional factor under low cell density by repressing HapR, preventing the activation of quorum sensing and ultimately reducing competitiveness during host infection [47]. Thus, pVa-22 may potentially modulate quorum-sensing pathways via PadR-like transcriptional regulators, which could contribute to attenuated virulence; however, this remains a preliminary hypothesis that requires further experimental validation.

Taken together, the results highlight the potential of pVa-22 as an antivirulence strategy. Although its bactericidal activity against *V. alginolyticus* in planktonic culture is limited, its ability to confer significant protection in the infection model underscores its therapeutic value. Such antivirulence strategies may offer an advantage over strictly lytic phages by preserving host-associated microbiota while selectively disarming pathogens. While antivirulence phages like pVa-22 offer ecological advantages, their practical application in aquaculture will require optimization of dosing, persistence under seawater conditions, and evaluation of resistance emergence, as well as further studies to elucidate the molecular basis of their antivirulence effects and assess their efficacy in aquaculture and other infection models.

## Supplemental Materials

Supplementary data for this paper are available on-line only at http://jmb.or.kr.



## Figures and Tables

**Table 1:** The host range of *Vibrio* phage pVa-22.